# Prognostic implication of leucocyte subpopulations in diffuse large B-cell lymphoma

**DOI:** 10.18632/oncotarget.17830

**Published:** 2017-05-12

**Authors:** Xiao Han, Jing Ruan, Wei Zhang, Daobin Zhou, Dongsheng Xu, Qiang Pei, Mingqi Ouyang, Mengxuan Zuo

**Affiliations:** ^1^ Department of Hematology, Peking Union Medical College Hospital, Beijing, China 100730; ^2^ Department of Hematopathology, CBLPath, Sonic Healthcare, Rye Brook, NY 10573, USA

**Keywords:** diffuse large B-cell lymphoma, prognosis, CD16- monocyte/CD16+ monocyte ratio, mature neutrophil/cytotoxic NK&T cell ratio, CytoDiff flow cytometric system

## Abstract

**Background:**

Recent studies have suggested that variables related to host adaptive immunity and the tumor microenvironment may predict the outcome in patients with non-Hodgkin's lymphoma. This study was undertaken to determine the prognostic value of peripheral blood leucocyte subpopulations in diffuse large-B-cell lymphoma patients.

**Methods:**

We prospectively analyzed the 16 leukocyte subpopulations using Cytodiff flow cytometric technique in a cohort of 45 diffuse large-B-cell lymphoma patients at a single institution between February and December 2014. The Cox proportional hazards model was used to evaluate prognostic factors for overall survival and progression free survival.

**Results:**

Diffuse large-B-cell lymphoma patients had decreased cytotoxic and non-cytotoxic NK&T cells as well as increased CD16+ monocytes, CD16- monocytes and mature neutrophils. The decreased CD16- monocyte/CD16+ monocyte ratio and increased mature neutrophil/cytotoxic NK&T cell ratio were related to poor progression-free and overall survival outcome in single and multivariate analysis. The co-constructed model using International Prognostic Index and mature neutrophil/cytotoxic NK&T cell ratio can also help discriminate the clinical outcome.

**Conclusions:**

The decreased CD16-monocyte/CD16+monocyte ratio and increased mature neutrophil/cytotoxic NK&T cell ratio predict poor prognosis in diffuse large-B-cell lymphoma patients. This finding provides a strong rationale for the study of cellular immunotherapy in B-cell lymphoma.

## INTRODUCTION

Diffuse large B-cell lymphoma (DLBCL) is the most common type of non-Hodgkin lymphoma (NHL). It is aggressive while potentially curable [1]. DLBCL is a heterogeneous entity with highly variable clinical outcomes [2]. The International Prognostic Index (IPI) and the Revised International Prognostic Index (R-IPI) are commonly used to evaluate the prognosis of DLBCL, even though they are still imprecise especially in the post-rituximab era [3, 4]. Therefore, other approaches are needed to help better identify high-risk DLBCL patients. Previous studies have shown that peripheral blood absolute lymphocyte count (ALC), absolute monocyte count (AMC) and the ALC/AMC ratio are related to clinical outcomes in DLBCL and classical Hodgkin lymphoma [5]. Recently, Park et al. demonstrated that lymphocyte subpopulations determined by the Cytodiff flow cytometric system are associated with the prognosis of metastatic gastric and lung cancers [5].

The Cytodiff flow cytometric system is able to automatically detect and separate 16 leukocyte subpopulations in the peripheral blood, including B lymphocytes, CD16+ cytotoxic NK&T lymphocytes, CD16- non-cytotoxic NK&T lymphocytes, CD16- monocytes, CD16+ monocytes, and neutrophils, generating a result called the extended leukocyte differential count [6]. The Cytodiff flow cytometric system has been recognized as a reliable and accurate way to count peripheral blood blasts [7–9]. It has also been used in research areas such as prognostic prediction of metastatic carcinoma, laboratory evaluation of sepsis severity [10] and monitoring of anti-viral therapy in HIV patients [11]. To the best of our knowledge, this detection system has not been used to evaluate the prognostic implication of changes in peripheral blood leukocyte subpopulations in NHL patients. In this prospective cohort study, we recruit 45 DLBCL patients and collect the 16 leukocyte subpopulations at the time of diagnosis to investigate the prognostic value of the leucocyte subpopulations.

## RESULTS

### Patient characteristics

There were 45 patients that met the study criteria and were included in this study. All 45 patients were first diagnosed with DLBCL without any treatment in PUMCH. The basic characteristics were presented in Table 1. RCHOP (rituximab, cyclophosphamide, doxorubicin, vincristine, prednisone) or RCHOP-like treatment was given to each patient. Five patients received autologous stem cell transplantation. As of January, 2016, the median follow-up was 16 months (range: 2-24months), the estimated 2-year overall survival (OS) was 0.763, and 2-year progression free survival (PFS) was 0.606 for the whole cohort. At the end of 16-month follow-up, 25 patients (55.6%) reached complete remission (CR), 8 (17.8%) reached partial remission (PR), 3 (6.7%) had progression disease (PD) and 9 (20%) died from disease progression. Eight patients had experienced disease relapse or refractory.

**Table 1:** Basic characteristics of diffuse B cell lymphoma patients

Characteristics	Number	Percentage(%)
Age		
<60	19	42.22
≥60	26	57.78
Sex		
Female	23	51.11
Male	22	48.89
Subtype		
DLBCL nos	35	77.78
DLBCL GCB	18	40.00
DLBCL ABC	17	37.78
PCNSL	4	8.89
ALK+DLBCL	1	2.22
DLBCL/BL	2	4.44
DLBCL of unknown	3	6.67
Ann Arbor Stage		
I, II	21	46.67
III, IV	24	53.33
B symptoms		
No	20	44.44
Yes	21	46.67
NA	4	8.89
Number of extra nodal sites		
0,1	29	64.44
>1	16	35.56
ECOG		
0,1	25	55.56
2,3,4	20	44.44
IPI		
0,1	19	42.22
2	10	22.22
3	6	13.33
4,5	10	22.22

Abbreviations: GCB DLBCL, germinal center B-cell-like diffuse large B-cell lymphoma; ABC DLBCL, activated B-cell-like diffuse large B-cell lymphoma; PCNSL, primary central nervous system lymphoma; ALK+DLBCL, anaplastic lymphoma kinase positive diffuse large B-cell lymphoma; DLBCL/BL, B-cell lymphoma, unclassifiable, with features intermediate between diffuse large B-cell lymphoma and Burkitt lymphoma; ECOG, Eastern Cooperative Oncology Group performance status; IPI, the international prognostic index.

### Leukocyte subpopulations are related to the prognosis of DLBCL

The numbers of leukocyte subsets and their differences between patients and healthy controls were shown in Table 2. The percentage and absolute number of all lymphocyte subpopulations including B lymphocytes, cytotoxic and non-cytotoxic NK&T cells in DLBCL patients significantly decreased compared to the controls. On the other hand, the patients had significantly greater relative and absolute mature neutrophils numbers as well as increased percentage of CD16+ monocytes while no significant difference was found in the absolute monocyte numbers. We performed single factor analysis comparing overall survival (OS) and progression-free survival (PFS) with all leukocyte subpopulations and other known prognostic variables including IPI (Supplementary Table 1). The decreased percentages of total NK&T lymphocytes, especially cytotoxic NK&T lymphocytes, were related to worse OS. Increased absolute numbers of both CD16+ and CD16- monocytes and the absolute number of mature neutrophils was associated to poor outcomes. In addition, all above changes except for the percentage of CD16+ monocytes were related to PFS. As for the well-known prognostic factors, only the number of extra-nodal sites, ECOG and IPI were found to be significantly related to patient outcomes.

**Table 2:** The level of subsets of leucocyte and their difference between patients and healthy controls

Clinical factors and leucocyte subsets	Controls		DLBCL patients		P value
	Mean	SD	Mean	SD	
Sex(male:female)	153:116		22:23		
Age(y)	41.95	13.49	60.89	11.02	*<0.05*
Lymphocytes(X10^9^)	2.07	0.61	1.37	0.78	*<0.001*
Lymphocytes(%)	33.96	7.20	21.99	12.69	*<0.001*
B lymphocytes(X10^9^)	0.21	0.09	0.14	0.17	*0.018*
B lymphocytes(%)	3.41	1.29	2.27	2.41	*0.003*
NK&T lymphocytes(X10^9^)	1.86	0.56	1.22	0.69	*<0.001*
NK&T lymphocytes(%)	30.54	6.77	19.72	11.51	*<0.001*
CD16+ cytotoxic NK&T lymphocytes(X10^9^)	0.50	0.27	0.28	0.25	*<0.001*
CD16+ cytotoxic NK&T lymphocytes(%)	8.29	3.94	4.26	3.23	*<0.001*
CD16- non-cytotoxic NK&T lymphocytes(X10^9^)	1.36	0.46	0.94	0.50	*<0.001*
CD16- non-cytotoxic NK&T lymphocytes(%)	22.25	5.68	15.45	9.67	*<0.001*
Monocytes(X10^9^)	0.41	0.11	0.56	0.71	0.183
Monocytes(%)	6.79	1.32	7.37	3.48	0.274
CD16- monocytes(X10^9^)	0.39	0.10	0.50	0.62	0.226
CD16- monocytes(%)	6.37	1.23	6.68	2.98	0.493
CD16+ monocytes(X10^9^)	0.03	0.02	0.06	0.10	0.061
CD16+ monocytes(%)	0.42	0.23	0.69	0.87	*0.049*
Mature neutrophils(X10^9^)	3.38	0.78	5.11	3.85	*0.004*
Mature neutrophils(%)	55.37	7.27	66.55	13.88	*<0.001*
Eosinophils(X10^9^)	0.17	0.11	0.13	0.17	*0.029*
Eosinophils(%)	2.77	1.55	2.04	2.63	*0.001*
Basophils(X10^9^)	0.05	0.02	0.06	0.20	0.618
Basophils(%)	0.80	0.39	0.67	1.02	0.408
Immature granulocytes(X10^9^)	0.01	0.01	0.12	0.51	0.141
Immature granulocytes(%)	0.19	0.16	0.89	2.03	*0.025*
CD16-monocytes/CD16+monocytes	19.05	10.15	16.63	15.08	0.172
Lymphocytes/monocytes	5.22	1.64	3.51	2.36	*<0.001*
Cytotoxic NK&T lymphocytes /CD16+ monocyte	24.46	18.14	10.43	8.18	*<0.001*
Cytotoxic NK&T lymphocytes /CD16- monocyte	1.37	0.75	0.74	0.60	*<0.001*
Mature neutrophils /monocyte	8.48	2.13	13.01	14.95	*0.049*
Mature neutrophils /CD16+monocyte	172.50	103.93	197.68	200.26	0.413
Mature neutrophils / cytotoxic NK&T lymphocytes	8.46	4.86	40.30	57.85	*0.001*
Mature neutrophils / noncytotoxic NK&T lymphocytes	2.73	1.04	8.86	14.67	*0.008*

Multivariate analysis was also analyzed for those factors with good performance in univariate analysis (Supplementary Table 2). Considering the sample size and event number of our study and the association between leucocyte subpopulations, the results of univariate analyses were adjusted by bone marrow involvement, IPI, ECOG and number of involved extra nodal sites. We found that only the absolute number of CD16+ monocytes and mature neutrophils were independently related to DLBCL outcomes (Table 3). As IPI is currently considered to be the most important clinical index for predicting DLBCL prognosis, we also constructed models that combined IPI with the absolute number of CD16+ monocytes or mature neutrophils (respectively) but the results accounting for IPI were insignificant.

**Table 3:** univariate and multivariate analysis for overall survival

Prognostic factors	Univariate analysis		Multivariate analysis^1^	
	HR^2^ (95% CI)	P value	HR (95%CI)	P value
Lymphocytes(%)	0.873(0.794-0.961)	0.005	0.917(0.823-1.021)	0.114
NK&T lymphocytes(%)	0.846(0.756-0.948)	0.004	0.896(0.787-1.021)	0.100
CD16- non-cytotoxic NK&T lymphocytes(%)	0.819(0.712-0.943)	0.005	0.882(0.752-1.033)	0.119
Monocytes(X10^9^)	16.75(2.413-116.3)	0.005	24.07(0.913-634.5)	0.057
CD16- monocytes(X10^9^)	42.58(2.236-810.9)	0.013	29.48(0.749-1160.5)	0.071
CD16+ monocytes(X10^9^)	5643 (74.33-4.3×105)	<0.001	2.53E9(11.27-5.67E17)	0.027
Mature neutrophils(X10^9^)	1.477(1.217-1.792)	<0.001	1.822(1.246-2.662)	0.002
Mature neutrophils(%)	1.07(1.008-1.135)	0.026	1.036(0.971-1.105)	0.280
CD16-monocytes/CD16+monocytes	0.862(0.761-0.976)	0.019	0.857(0.759-0.967)	0.012
Cytotoxic NK&T lymphocytes /CD16+ Monocyte	0.859(0.747-0.986)	0.031	0.907(0.791-1.039)	0.160
Mature neutrophils/cytotoxic NK&T lymphocytes	1.016 (1.006-1.025)	0.001	1.018(1.003-1.034)	0.019
Mature neutrophils/noncytotoxic NK&T lymphocytes	1.03 (1.009-1.051)	0.006	0.983(0.931-1.037)	0.526

^1^Multivariate analysis was adjusted by IPI, ECOG, number of extra nodal involvement and bone marrow.

^2^HR: Hazard Ratio

### CD16- monocyte/CD16+ monocyte and mature neutrophil/cytotoxic NK&T cell ratios can predict the prognosis of DLBCL with high specificity

Several ratios between the leukocyte subsets were evaluated for OS and PFS with cox analysis. Decreased CD16-/CD16+ monocyte ratio and increased mature neutrophil/cytotoxic NK&T cell ratio were found to be associated with shorter overall survival time in both single-factor and multi-factor analysis. Mature neutrophil/cytotoxic NK&T cell ratio was also related to PFS in Cox analysis (Table 2).

**Figure 1:**
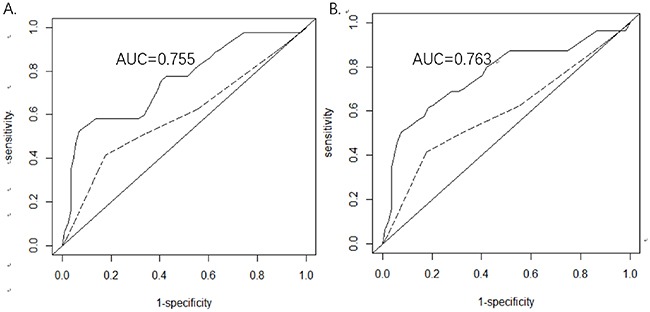
(A) The receiver operating characteristic (ROC) curve of 2-year-survival based on the CD16-monocytes/CD16+monocyte ratio **(B)** The ROC curve of 2-year-survival based on mature neutrophils/cytotoxic NK&T cell ratio. The dashed line represents the ROC curve of 2-year-survival for IPI. Abbreviations: AUC, area under ROC curve.

To further determine the prognostic value of CD16-/CD16+ monocyte ratio and mature neutrophil/cytotoxic NK&T cell ratio, the time-dependent receiver operating characteristic (ROC) curve of 2-year-survival was performed (Figure 1). The CD16-/CD16+ monocyte ratio seemed to be the most specific index to predict the overall survival of DLBCL patients. Using a cut-off value of 7.5 resulted in diagnostic specificity being 93% and sensitivity being 52% (area under curve (AUC) = 0.755). A mature neutrophil/cytotoxic NK&T cell ratio cut-off value of 46 resulted in diagnostic sensitivity of 50% and specificity of 93% (AUC = 0.763). The time-dependent AUCs of CD16- monocyte/CD16+ monocyte ratio and neutrophil/cytotoxic NK&T cell ratios were both significantly higher than IPI in our study (p<0.01).

**Figure 2:**
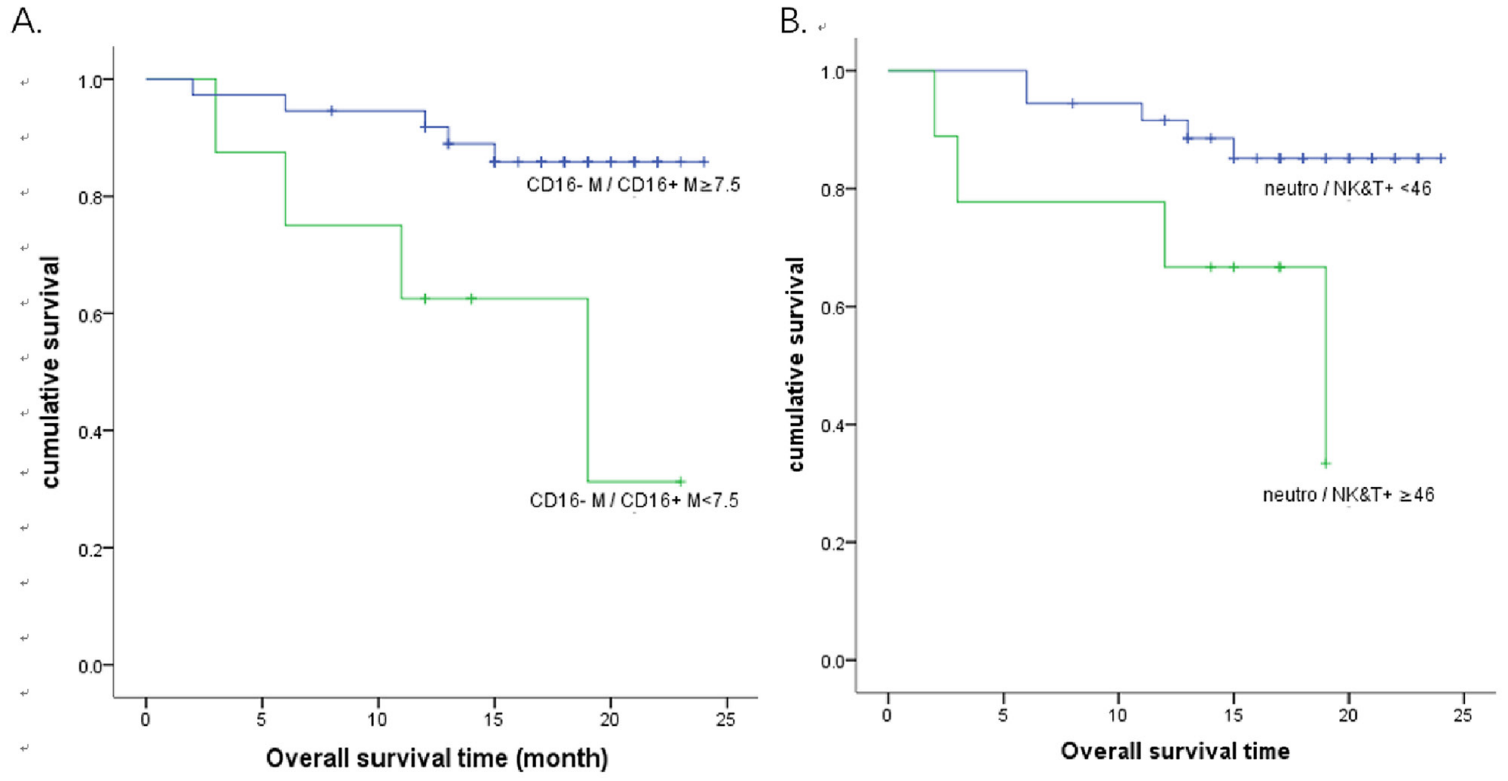
Kaplan-Meier survival curves for overall survival **(A)** The estimated 2-year overall survival rate was 0.313 and 0.859 for CD16- monocyte/CD16+ monocyte ratio <7.5 and ≥7.5, respectively (p<0.05). **(B)** The estimated 2-year overall survival rate was 0.851 and 0.333 for mature neutrophil/cytotoxic NK&T cell ratio of <46 and ≥46, respectively (p<0.05).

The Kaplan–Meier analysis of overall survival was also performed for these two ratios with the cut-off value determined by the survival ROC analysis. The estimated 2-year overall survival rate was 0.313 and 0.859 for CD16-/CD16+ monocyte ratio <7.5 and ≥7.5, respectively (p<0.05). For mature neutrophil/cytotoxic NK&T cell ratio of <46 and ≥46, the 2-year overall survival rate was 0.851 and 0.333, respectively (p<0.05) (Figure 2).

**Figure 3:**
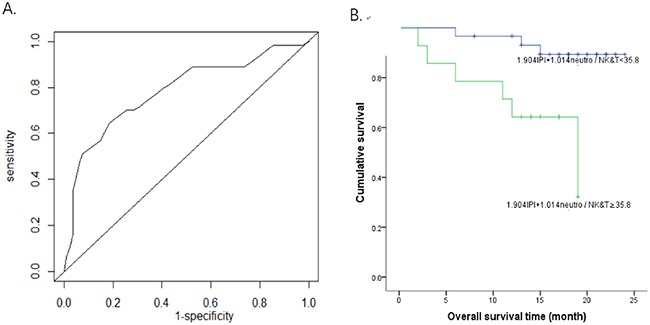
Survival analysis using a model constructed by mature neutrophils/cytotoxic NK&T cells and IPI **(A)** The survival receiver operating characteristic (ROC) curve of 2-year-survival for mature neutrophils/cytotoxic NK&T cells and IPI model. AUC was 0.781. **(B)** The overall survival curve for 1.904IPI+1.014 mature neutrophils/cytotoxic NK&T cells <35.8 and ≥35.8 showed the 2 year overall survival rate was 0.894 and 0.321 respectively in the two groups (p=0.004).

Finally, we evaluated models that combined IPI with either CD16-/CD16+ monocyte ratio or mature neutrophil/cytotoxic NK&T cell ratio for estimating the overall survival of patients with DLBCL (Table 4). We found that the model constructed using IPI and CD16-/CD16+ monocyte ratio gave an AUC of 0.702, with the cutoff value of 13, there is no statistical significance in Kaplan–Meier analysis (p = 0.115). In contrast, the model combining IPI with mature neutrophil/cytotoxic NK&T cell ratio showed great performance with an AUC of 0.781 and 2-year overall survival rate of 0.321 and 0.894 in the two groups respectively (p=0.004, Figure 3).

**Table 4:** Prognostic models for overall survival constructed with IPI, CD16-/CD16+ monocyte ratio, and mature neutrophil/cytotoxic NK&T cell ratio

	β	95% CI	P value
**Model 1**			
IPI	2.025	1.114-3.683	0.021
CD16-M/CD16+M	0.887	0.788-0.998	0.048
**Model 2**			
IPI	1.904	1.068-3.397	0.029
Neutro/ NK&T	1.014	1.004-1.024	0.008

Abbreviations: C16-M/CD16+M, CD16- monocytes/CD16+ monocytes; Neutro/ NK&T, mature neutrophils /cytotoxic NK&T lymphocytes.

## DISCUSSION

Previous studies have shown that low absolute lymphocytes and NK cells, along with increased monocytes and mature neutrophils all indicate a poor outcome and shorter OS and/or PFS [12–18]. However, the cytotoxic T cells play an uncertain role in predicting the survival of patients with DLBCL [19–21]. The current study aims to further elucidate the roles of peripheral leukocytes in prognosis of DLBCL by investigating the prognostic significance of changes in 16 leukocyte subpopulations, including the subsets of lymphocytes and monocytes.

We demonstrated that the DLBCL patients had decreased number and percentage of all lymphocyte subsets. This phenomenon may suggest an immune defect in DLBCL patients [21] or the antitumor response to a high tumor burden [17]. In addition, there is an increase in mature neutrophils and the percentage of CD16+ monocytes, which may be due to tumor-derived chemokines that can induce proliferation of neutrophils and monocytes, thus promoting tumor growth [22–24]. Furthermore, we are the first to demonstrate that CD16-monocyte/CD16+monocyte and mature neutrophil/cytotoxic NK&T cell ratios are both independent indicators for OS in DLBCL patients based on multi-factor and ROC analyses. These ratios may provide additional prognostic information when used in conjunction with the International Prognostic Index in the post-rituximab era.

It has been shown that increased neutrophil/lymphocyte ratio at diagnosis of DLBCL independently represents poor prognosis [25, 26]. The lymphocyte/monocyte ratio [5, 27–29] is also a useful prognostic indicator for DLBCL patients in several studies. The lymphocyte/monocyte ratio was also tested in our study. No statistical significant results were found which may due to different populations or insufficient samples.

According to the 2010 Nomenclature Committee of the International Union of Immunological Societies, monocytes are divided into 3 types [30], the classical CD14++CD16- monocytes (about 91% of the blood monocytes), intermediate CD14+CD16+ cells and non-classical CD14+CD16++ monocytes [31]. The latter two were previously called CD16+ monocytes. Once emigrated to the target tissue, circulating monocytes differentiate into macrophages and polarize into distinct subtypes depending on the microenvironment [24] While the classical monocytes have high phagocytic and antimicrobial activity, CD16+ monocytes have been shown to participate in anti-tumor responses [32]. However, in some disease states, circulating CD16+ monocytes in peripheral blood have been shown to promote tumor growth [33] and indicate the worse prognosis. In current study, we found a small increase in CD16-monocytes and a greater (1.8-fold on average) increase in CD16+monocytes in DLBCL patients compared to healthy controls. The decreased CD16-monocyte/CD16+monocyte ratio is associated with poor prognosis. Subimerb et al found that in cholangiocarcinoma patients, the peripheral CD16+ monocytes had tumor-promoting characteristics and expressed higher levels of growth factor (EREG) and angiogenic chemokines (CXCL3), these cytokines corresponded to a M2 pro-tumorigenic phenotype [34]. M2-like macrophages have been shown to develop pro-angiogenic functions and promote angiogenesis which is associated with poor OS in NHL including DLBCL in several studies [35–37]. It is possible that with increased circulating CD16+ monocytes, more M2-like macrophages may infiltrate the tumor microenvironment and promote angiogenesis while suppressing host antitumor immunity, resulting in worsened clinical outcomes.

We have also shown that an increased mature neutrophil/cytotoxic NK&T cell ratio was associated with poor prognosis. Two factors may contribute to this observation including either increase in neutrophils or the decrease in NK&T cells. The increased inflammatory cells including mature neutrophils can release growth and survival factors, stimulating DNA damage, promoting angiogenesis and tumor evasion of the host defense system [38, 39]. On the other hand, the majority of the CD16+ cytotoxic NK&T cells are NK cells according to the Cytodiff system [40], which are important effectors in antitumor immunity [41–43]. Studies have shown that low NK cells are associated with worse prognosis in DLBCL patients [17].

Our study has some limitations. The predictive value of these ratios for overall survival and progression-free survival and the dynamic change of the ratios during disease improvement or progression and relapse should also be further confirmed in a larger population and a longer follow-up. Regular white blood cell subsets measurement should be done in the future to examine whether effective treatment can normalize the white blood cell subsets. Furthermore, the NK cells and T cells cannot be separated by this system and restricts further investigation into the roles played by these immune cells.

In conclusion, we are the first to demonstrate that the CD16-monocyte/CD16+monocyte and mature neutrophil/cytotoxic NK&T cell ratios are valuable indices to discriminate the overall survival of DLBCL patients in post-rituximab era. This study may also help us better understand the mechanism especially the roles of host immune homeostasis and tumor microenvironment in lymphoma development and progression.

## MATERIALS AND METHODS

### Patient and controls

Patients diagnosed with DLBCL, according to the 2008 World Health Organization (WHO) classification of hematopoietic malignancies [44], at Peking Union Medical College Hospital (PUMCH) between February and December 2014 were included in this study. All patients were over 18 years old and were first diagnosed without any prior treatment. Pregnant patients were excluded. 269 healthy volunteers without known diseases aged 18-80 years old were also recruited from the physical examination center of PUMCH at the same time. All patients and volunteers provided written informed consent and the study was approved by the Ethics Committee of Peking Union Medical College Hospital.

### Data collection and follow-up

The following parameters were collected: age, sex, subtype, Eastern Cooperative Oncology Group (ECOG) performance status (PS), Ann Arbor stage (I-IV), absence or presence of B symptoms, number and type of involved sites, prognostic index including International Prognostic Index (IPI) for DLBCL based on medical record review. The peripheral blood from all 45 patients and 269 controls were also collected for further flow cytometric analysis upon admission in the study. All patients received regular R-CHOP treatment and follow-up in our facility. During the follow up, treatment response was evaluated by enhanced computed tomography or PET-CT. Complete remission (CR), relapse and progression of lymphoma were evaluated according to previously published reports [45, 46].

### Flow cytometry analysis

The extended blood leukocyte differential count was determined using the flow cytometer (FC500) in conjunction with premixed CytoDiff™ reagent and analysis software (Beckman Coulter, USA). Antibodies used in CytoDiff™ include CD36-FITC, CD2-PE, CD294-PE, CD19-ECD, CD16-PC5, and CD45-PC7. The leukocytes were differentiated into 16 cell populations (B-lymphocytes, CD16- T-lymphocytes, CD16+ T and NK cells, T and NK lymphocytes, total lymphocytes, CD16 monocytes, CD16+ monocytes, total monocytes, immature granulocytes [IGs], total eosinophil, mature neutrophils, total neutrophils, B blasts, T blasts, non-B-non-T blasts, and total basophils). Analysis procedures were conducted according to the manufacturer's instructions. Briefly, 100 μL of whole blood samples was mixed with 10 μL of CytoDiff reagent. After 20 minutes of incubation at room temperature, the red blood cells were then lysed by Versalyse solution (Beckman Coulter) for 15 min. Approximately 20,000 cells were acquired and analyzed automatically by the analysis software. The analysis software is self-gating and separates populations by automatic logic pathways. [6]

### Statistical analysis

The correlation between the possible prognostic value and clinical parameters was assessed by the chi-square test. Progression-free survival (PFS) and overall survival (OS) were estimated using the Kaplan–Meier method and two-tailed log-rank test. The Cox proportional hazards model was used to evaluate the lymphocyte subtypes as prognostic factors for PFS and OS and to adjust for other known prognostic variables included in the IPI. All two-sided P values <0.05 were considered to be statistically significant. All these statistical tests were carried out using SPSS 19.0 software (SPSS Inc., Chicago. USA).

Specificity, sensitivity and cut-off were established using time-dependent receiver operating characteristic (ROC) curve analysis. As proposed by previous publication [47], we considered that area under curve (AUC) values >0.7 indicate that the parameter can be used for diagnosis, with values >0.9 indicating high clinical accuracy. We used R software version 3.3.1 including the “time ROC” package and the “survival ROC” package to do the time-dependent AUC comparison and ROC curve analysis [48].

## SUPPLEMENTARY MATERIALS TABLES







## References

[1] Habermann TM (2012). New developments in the management of diffuse large B-cell lymphoma. Hematology.

[2] Gisselbrecht C, Glass B, Mounier N, Singh Gill D, Linch DC, Trneny M, Bosly A, Ketterer N, Shpilberg O, Hagberg H, Ma D, Brière J, Moskowitz CH, Schmitz N (2010). Salvage regimens with autologous transplantation for relapsed large B-cell lymphoma in the rituximab era. J Clin Oncol.

[3] Panizo C, Rodríguez AJ, Gutiérrez G, Díaz FJ, González-Barca E, de Oña R, Grande C, Sancho JM, García-Álvarez MF, Sánchez-González B, Peñalver FJ, Cannata J, Espeso M (2015). Evaluation of clinical and biological prognostic factors in relapsed or refractory diffuse large B-cell lymphoma patients after previous treatment with rituximab and chemotherapy: results of the PRO-R-IPI study. Clin Lymphoma Myeloma Leuk.

[4] Huang YC, Liu CY, Lu HJ, Liu HT, Hung MH, Hong YC, Hsiao LT, Gau JP, Liu JH, Hsu HC, Chiou TJ, Chen PM, Tzeng CH, Yu YB (2013). Comparison of prognostic models for patients with diffuse large B-cell lymphoma in the rituximab era. Ann Hematol.

[5] Porrata LF, Ristow KM, Habermann TM, Witzig TE, Colgan JP, Inwards DJ, Ansell SM, Micallef IN, Johnston PB, Nowakowski G, Thompson CA, Markovic SN (2014). Peripheral blood absolute lymphocyte/monocyte ratio during rituximab, cyclophosphamide, doxorubicin, vincristine and prednisone treatment cycles predicts clinical outcomes in diffuse large B-cell lymphoma. Leuk Lymphoma.

[6] Faucher JL, Lacronique-Gazaille C, Frébet E, Trimoreau F, Donnard M, Bordessoule D, Lacombe F, Feuillard J (2007). “6 markers/5 colors” extended white blood cell differential by flow cytometry. Cytometry A.

[7] Kahng J, Kim Y, Kim M, Oh EJ, Park YJ, Han K (2015). Flow cytometric white blood cell differential using CytoDiff is excellent for counting blasts. Ann Lab Med.

[8] Gac F, Thibert JB, Le Berre C, Le Priol J, Semana G, Fest T, Roussel M (2013). Evaluation of CytoDiff™ on cord blood WBC differential. Int J Lab Hematol.

[9] Kim AH, Lee W, Kim M, Kim Y, Han K (2014). White blood cell differential counts in severely leukopenic samples: a comparative analysis of different solutions available in modern laboratory hematology. Blood Res.

[10] Park SH, Park BG, Park CJ, Kim S, Kim DH, Jang S, Hong SK, Chi HS (2014). An extended leukocyte differential count (16 types of circulating leukocytes) using the CytoDiff flow cytometric system can provide information for the discrimination of sepsis severity and prediction of outcome in sepsis patients. Cytometry B Clin Cytom.

[11] Kviatkovskaia SV, Sukhacheva EA, Reshetnikov IV, Dolgova NV, Tseĭlikman OB, Tseĭlikman VE (2014). [The application of panel CytoDiff for monitoring of effectiveness of antiviral therapy in HIV-infected patients]. [Article in Russian]. Klin Lab Diagn.

[12] Balkwill F, Mantovani A (2001). Inflammation and cancer: back to Virchow?. Lancet.

[13] Cox MC, Nofroni I, Laverde G, Ferrari A, Amodeo R, Tatarelli C, Saltarelli F, Veggia B, Aloe-Spiriti MA, Ruco L, Monarca B (2008). Absolute lymphocyte count is a prognostic factor in diffuse large B-cell lymphoma. Br J Haematol.

[14] Cox MC, Nofroni I, Ruco L, Amodeo R, Ferrari A, La Verde G, Cardelli P, Montefusco E, Conte E, Monarca B, Aloe-Spiriti MA (2008). Low absolute lymphocyte count is a poor prognostic factor in diffuse-large-B-cell-lymphoma. Leuk Lymphoma.

[15] Moore MM, Chua W, Charles KA, Clarke SJ (2010). Inflammation and cancer: causes and consequences. Clin Pharmacol Ther.

[16] Wilcox RA, Ristow K, Habermann TM, Inwards DJ, Micallef IN, Johnston PB, Colgan JP, Nowakowski GS, Ansell SM, Witzig TE, Markovic SN, Porrata L (2011). The absolute monocyte and lymphocyte prognostic score predicts survival and identifies high-risk patients in diffuse large-B-cell lymphoma. Leukemia.

[17] Plonquet A, Haioun C, Jais JP, Debard AL, Salles G, Bene MC, Feugier P, Rabian C, Casasnovas O, Labalette M, Kuhlein E, Farcet JP, Emile JF (2007). Peripheral blood natural killer cell count is associated with clinical outcome in patients with aaIPI 2-3 diffuse large B-cell lymphoma. Ann Oncol.

[18] Ansell SM, Stenson M, Habermann TM, Jelinek DF, Witzig TE (2001). Cd4+ T-cell immune response to large B-cell non-Hodgkin's lymphoma predicts patient outcome. J Clin Oncol.

[19] Hasselblom S, Sigurdadottir M, Hansson U, Nilsson-Ehle H, Ridell B, Andersson PO (2007). The number of tumour-infiltrating TIA-1+ cytotoxic T cells but not FOXP3+ regulatory T cells predicts outcome in diffuse large B-cell lymphoma. Br J Haematol.

[20] Muris JJ, Meijer CJ, Cillessen SA, Vos W, Kummer JA, Bladergroen BA, Bogman MJ, MacKenzie MA, Jiwa NM, LH Siegenbeek van Heukelom, Ossenkoppele GJ, Oudejans JJ (2004). Prognostic significance of activated cytotoxic T-lymphocytes in primary nodal diffuse large B-cell lymphomas. Leukemia.

[21] Rimsza LM, Roberts RA, Miller TP, Unger JM, LeBlanc M, Braziel RM, Weisenberger DD, Chan WC, Muller-Hermelink HK, Jaffe ES, Gascoyne RD, Campo E, Fuchs DA (2004). Loss of MHC class II gene and protein expression in diffuse large B-cell lymphoma is related to decreased tumor immunosurveillance and poor patient survival regardless of other prognostic factors: a follow-up study from the Leukemia and Lymphoma Molecular Profiling Project. Blood.

[22] Chang C, Wu SY, Kang YW, Lin KP, Chen TY, Medeiros LJ, Chang KC (2015). High levels of regulatory T cells in blood are a poor prognostic factor in patients with diffuse large B-cell lymphoma. Am J Clin Pathol.

[23] Fridlender ZG, Sun J, Kim S, Kapoor V, Cheng G, Ling L, Worthen GS, Albelda SM (2009). Polarization of tumor-associated neutrophil phenotype by TGF-beta: “N1” versus “N2” TAN. Cancer Cell.

[24] Sica A, Larghi P, Mancino A, Rubino L, Porta C, Totaro MG, Rimoldi M, Biswas SK, Allavena P, Mantovani A (2008). Macrophage polarization in tumour progression. Semin Cancer Biol.

[25] Porrata LF, Ristow K, Habermann T, Inwards DJ, Micallef IN, Markovic SN (2010). Predicting survival for diffuse large B-cell lymphoma patients using baseline neutrophil/lymphocyte ratio. Am J Hematol.

[26] Troppan K, Deutsch A, Gerger A, Stojakovic T, Beham-Schmid C, Wenzl K, Neumeister P, Pichler M (2014). The derived neutrophil to lymphocyte ratio is an independent prognostic factor in patients with diffuse large B-cell lymphoma. Br J Cancer.

[27] Rambaldi A, Boschini C, Gritti G, Delaini F, Oldani E, Rossi A, Barbui AM, Caracciolo D, Ladetto M, Gueli A, De Crescenzo A, Passera R, Devizzi L (2013). The lymphocyte to monocyte ratio improves the IPI-risk definition of diffuse large B-cell lymphoma when rituximab is added to chemotherapy. Am J Hematol.

[28] Li YL, Pan YY, Jiao Y, Ning J, Fan YG, Zhai ZM (2014). Peripheral blood lymphocyte/monocyte ratio predicts outcome for patients with diffuse large B cell lymphoma after standard first-line regimens. Ann Hematol.

[29] Li YL, Gu KS, Pan YY, Jiao Y, Zhai ZM (2014). Peripheral blood lymphocyte/monocyte ratio at the time of first relapse predicts outcome for patients with relapsed or primary refractory diffuse large B-cell lymphoma. BMC Cancer.

[30] Ziegler-Heitbrock L, Ancuta P, Crowe S, Dalod M, Grau V, Hart DN, Leenen PJ, Liu YJ, MacPherson G, Randolph GJ, Scherberich J, Schmitz J, Shortman K (2010). Nomenclature of monocytes and dendritic cells in blood. Blood.

[31] Abeles RD, McPhail MJ, Sowter D, Antoniades CG, Vergis N, Vijay GK, Xystrakis E, Khamri W, Shawcross DL, Ma Y, Wendon JA, Vergani D (2012). CD14, CD16 and HLA-DR reliably identifies human monocytes and their subsets in the context of pathologically reduced HLA-DR expression by CD14(hi) /CD16(neg) monocytes: expansion of CD14(hi) /CD16(pos) and contraction of CD14(lo) /CD16(pos) monocytes in acute liver failure. Cytometry A.

[32] Sulicka J, Surdacki A, Mikołajczyk T, Strach M, Gryglewska B, Ćwiklińska M, Balwierz W, Guzik T, Grodzicki TK (2013). Elevated markers of inflammation and endothelial activation and increased counts of intermediate monocytes in adult survivors of childhood acute lymphoblastic leukemia. Immunobiology.

[33] De Palma M, Venneri MA, Galli R, Sergi Sergi L, Politi LS, Sampaolesi M, Naldini L (2005). Tie2 identifies a hematopoietic lineage of proangiogenic monocytes required for tumor vessel formation and a mesenchymal population of pericyte progenitors. Cancer Cell.

[34] Subimerb C, Pinlaor S, Lulitanond V, Khuntikeo N, Okada S, McGrath MS, Wongkham S (2010). Circulating CD14(+) CD16(+) monocyte levels predict tissue invasive character of cholangiocarcinoma. Clin Exp Immunol.

[35] Gratzinger D, Zhao S, Tibshirani RJ, Hsi ED, Hans CP, Pohlman B, Bast M, Avigdor A, Schiby G, Nagler A, Byrne GE, Lossos IS, Natkunam Y (2008). Prognostic significance of VEGF, VEGF receptors, and microvessel density in diffuse large B cell lymphoma treated with anthracycline-based chemotherapy. Lab Invest.

[36] Ruan J, Hajjar K, Rafii S, Leonard JP (2009). Angiogenesis and antiangiogenic therapy in non-Hodgkin's lymphoma. Ann Oncol.

[37] Farinha P, Masoudi H, Skinnider BF, Shumansky K, Spinelli JJ, Gill K, Klasa R, Voss N, Connors JM, Gascoyne RD (2005). Analysis of multiple biomarkers shows that lymphoma-associated macrophage (LAM) content is an independent predictor of survival in follicular lymphoma (FL). Blood.

[38] de Visser KE, Eichten A, Coussens LM (2006). Paradoxical roles of the immune system during cancer development. Nat Rev Cancer.

[39] Finn OJ (2012). Immuno-oncology: understanding the function and dysfunction of the immune system in cancer. Ann Oncol.

[40] Roussel M, Benard C, Ly-Sunnaram B, Fest T (2010). Refining the white blood cell differential: the first flow cytometry routine application. Cytometry A.

[41] Wu J, Lanier LL (2003). Natural killer cells and cancer. Adv Cancer Res.

[42] Brenner CD, King S, Przewoznik M, Wolters I, Adam C, Bornkamm GW, Busch DH, Röcken M, Mocikat R (2010). Requirements for control of B-cell lymphoma by NK cells. Eur J Immunol.

[43] Street SE, Hayakawa Y, Zhan Y, Lew AM, MacGregor D, Jamieson AM, Diefenbach A, Yagita H, Godfrey DI, Smyth MJ (2004). Innate immune surveillance of spontaneous B cell lymphomas by natural killer cells and gammadelta T cells. J Exp Med.

[44] Swerdlow SH, Campo E, Harris NL, Jaffe ES, Pileri SA, Stein H, Thiele J, Vardiman JW (2008). WHO Classification of Tumours of Haematopoietic and Lymphoid Tissues.

[45] Cheson BD, Horning SJ, Coiffier B, Shipp MA, Fisher RI, Connors JM, Lister TA, Vose J, Grillo-López A, Hagenbeek A, Cabanillas F, Klippensten D, Hiddemann W (1999). Report of an international workshop to standardize response criteria for non-Hodgkin's lymphomas. J Clin Oncol.

[46] Cheson BD, Pfistner B, Juweid ME, Gascoyne RD, Specht L, Horning SJ, Coiffier B, Fisher RI, Hagenbeek A, Zucca E, Rosen ST, Stroobants S, Lister TA (2007). Revised response criteria for malignant lymphoma. J Clin Oncol.

[47] Swets JA (1982). Evaluation of diagnostic systems: methods for signal detection theory.

[48] Heagerty PJ, Lumley T, Pepe MS (2000). Time-dependent ROC curves for censored survival data and a diagnostic marker. Biometrics.

