# Supplementary material for: Prognostic implication of leucocyte subpopulations in diffuse large B-cell lymphoma
Source: Oncotarget. 2017 May 12;8(29):47790–800. doi: 10.18632/oncotarget.17830 (PMC5564605; doi:10.18632/oncotarget.17830)
Supplement: Supplementary file 1 [file oncotarget-08-47790-s001.pdf]

Prognostic implication of leucocyte subpopulations in diffuse large B-cell lymphoma

SUPPLEMENTARY TABLES

Supplementary Table 1: Hazard ratios of subsets of clinical characteristics and leukocytes subsets for OS and PFS evaluation with cox analysis.

See Supplementary File 1

Supplementary Table 2: Multivariate analysis for overall survival.

See Supplementary File 2
