# Supplementary material for: Prognostic implication of leucocyte subpopulations in diffuse large B-cell lymphoma
Source: Oncotarget. 2017 May 12;8(29):47790–800. doi: 10.18632/oncotarget.17830 (PMC5564605; doi:10.18632/oncotarget.17830)
Supplement: Supplementary file 2 [file oncotarget-08-47790-s002.docx]

**S1 Table. Hazard ratios of subsets of clinical characteristics and leukocytes subsets for OS and PFS evaluation with cox analysis**

| \| **Prognostic factors** \| \| **Overall survival (OS)** \| \| \| **Progression-free survival (PFS)** \| \| \| \| --- \| --- \| --- \| --- \| --- \| --- \| --- \| --- \| \| HR \| 95% CI \| p \| HR \| 95% CI \| p \| \| Age（≥60y vs <60y） \| \| 1.022 \| 0.274-3.809 \| 0.975 \| 1.092 \| 0.378-3.153 \| 0.87 \| \| Sex（male vs female） \| \| 2.367 \| 0.590-9.497 \| 0.224 \| 1.559 \| 0.539-4.510 \| 0.412 \| \| Stage（III,IV vs I, II） \| \| 3.164 \| 0.656-15.28 \| 0.152 \| 2.466 \| 0.770-7.904 \| 0.129 \| \| B symptoms (yes vs no) \| \| 1.993 \| 0.474-8.379 \| 0.347 \| 1.431 \| 0.480-4.270 \| 0.52 \| \| Number of extra nodal sites (>1 vs 0,1) \| \| 4.483 \| 1.117-18 \| 0.034 \| 3.157 \| 1.088-9.159 \| 0.034 \| \| ECOG (2,3,4 vs 0,1) \| \| 5.495 \| 1.137-26.56 \| 0.034 \| 2.141 \| 0.740-6.193 \| 0.16 \| \| IPI (3,4,5 vs 0,1,2) \| \| 4.887 \| 1.206-19.80 \| 0.026 \| 3.548 \| 1.219-10.32 \| 0.02 \| \| lymphocytes(X109) \| \| 0.58 \| 0.200-1.681 \| 0.316 \| 0.566 \| 0.24-1.333 \| 0.193 \| \| lymphocytes(%) \| \| 0.873 \| 0.794-0.961 \| 0.005 \| 0.915 \| 0.860-0.974 \| 0.006 \| \| B lymphocytes(X109) \| \| 0.089 \| 0-32.107 \| 0.42 \| 0.021 \| 0-4.738 \| 0.163 \| \| B lymphocytes(%) \| \| 0.694 \| 0.430-1.122 \| 0.136 \| 0.667 \| 0.449-0.991 \| 0.045 \| \| NK&T lymphocytes(X10^9^) \| \| 0.551 \| 0.161-1.881 \| 0.341 \| 0.563 \| 0.213-1.492 \| 0.248 \| \| NK&T lymphocytes(%) \| \| 0.846 \| 0.756-0.948 \| 0.004 \| 0.906 \| 0.842-0.974 \| 0.008 \| \| CD16+ cytotoxic NK&T lymphocytes(X10^9^) \| \| 0.325 \| 0.010-10.118 \| 0.522 \| 0.442 \| 0.033-6.015 \| 0.54 \| \| CD16+ cytotoxic NK&T lymphocytes(%) \| \| 0.705 \| 0.492-1.010 \| 0.056 \| 0.825 \| 0.652-1.043 \| 0.108 \| \| CD16- non-cytotoxic NK&T lymphocytes(X10^9^) \| \| 0.455 \| 0.094-2.195 \| 0.327 \| 0.44 \| 0.124-1.559 \| 0.203 \| \| CD16- non-cytotoxic NK&T lymphocytes(%) \| \| 0.819 \| 0.712-0.943 \| 0.005 \| 0.886 \| 0.808-0.972 \| 0.01 \| \| monocytes(X10^9^) \| \| 16.75 \| 2.413-116.3 \| 0.004 \| 14.56 \| 2.622-80.87 \| 0.002 \| \| monocytes(%) \| \| 1.038 \| 0.864-1.247 \| 0.689 \| 1.06 \| 0.926-1.213 \| 0.4 \| \| CD16- monocytes(X10^9^) \| \| 42.578 \| 2.236-810.9 \| 0.013 \| 39.13 \| 2.977-514.3 \| 0.005 \| \| CD16- monocytes(%) \| \| 0.982 \| 0.769-1.254 \| 0.885 \| 1.042 \| 0.885-1.226 \| 0.62 \| \| CD16+ monocytes(X10^9^) \| \| 5643.89 \| 74.33-4.3X10^5^ \| <0.001 \| 2682 \| 50.93-1.4X10^5^ \| <0.001 \| \| CD16+ monocytes(%) \| \| 1.634 \| 1.029-2.595 \| 0.037 \| 1.5 \| 0.963-2.335 \| 0.073 \| \| mature neutrophils(X10^9^) \| \| 1.477 \| 1.217-1.792 \| <0.001 \| 1.405 \| 1.187-1.663 \| <0.001 \| \| mature neutrophils(%) \| \| 1.07 \| 1.008-1.135 \| 0.026 \| 1.049 \| 1.004-1.096 \| 0.033 \| \| eosinophils(X10^9^) \| \| 0.003 \| 0-42.473 \| 0.233 \| 0.001 \| 0-2.043 \| 0.074 \| \| eosinophils(%) \| \| 0.506 \| 0.249-1.027 \| 0.059 \| 0.522 \| 0.305-0.893 \| 0.018 \| \| basophils(X10^9^) \| \| 6.471 \| 1.146-36.55 \| 0.035 \| 6.296 \| 1.099-36.062 \| 0.039 \| \| basophils(%) \| \| 1.363 \| 0.874-2.127 \| 0.172 \| 1.319 \| 0.829-2.098 \| 0.242 \| \| immature granulocytes(X10^9^) \| \| 20.02 \| 0.727-551.3 \| 0.076 \| 16.11 \| 1.001-259.3 \| 0.05 \| \| immature granulocytes(%) \| \| 1.314 \| 1.039-1.663 \| 0.023 \| 1.37 \| 1.119-1.677 \| 0.002 \| \| CD16-monocytes/CD16+monocytes \| \| 0.862 \| 0.761-0.976 \| 0.019 \| 0.959 \| 0.892-1.031 \| 0.033 \| \| lymphocytes/monocytes \|  \| 0.68 \| 0.418-1.105 \| 0.119 \| 0.635 \| 0.418-0.964 \| 0.033 \| \| cytotoxic NK&T lymphocytes /CD16+ monocyte \| \| 0.859 \| 0.747-0.986 \| 0.031 \| 0.881 \| 0.795-0.976 \| 0.015 \| \| cytotoxic NK&T lymphocytes /CD16- monocyte \| \| 0.502 \| 0.106-2.375 \| 0.384 \| 0.436 \| 0.118-1.616 \| 0.214 \| \| mature neutrophils /monocyte \| \| 1.03 \| 0.997-1.060 \| 0.08 \| 1.02 \| 0.991-1.051 \| 0.17 \| \| mature neutrophils /CD16+monocyte \| \| 0.996 \| 0.990-1.003 \| 0.301 \| 0.999 \| 0.996-1.002 \| 0.692 \| \| mature neutrophils /cytotoxic NK&T lymphocytes \| \| 1.016 \| 1.006-1.025 \| 0.001 \| 1.017 \| 1.009-1.026 \| <0.001 \| \| mature neutrophils /noncytotoxic NK&T lymphocytes \| \| 1.03 \| 1.009-1.051 \| 0.006 \| 1.024 \| 1.005-1.044 \| 0.013 \| |  |  |  |  |  |  |  |
| --- | --- | --- | --- | --- | --- | --- | --- | --- | --- | --- | --- | --- | --- | --- | --- | --- | --- | --- | --- | --- | --- | --- | --- | --- | --- | --- | --- | --- | --- | --- | --- | --- | --- | --- | --- | --- | --- | --- | --- | --- | --- | --- | --- | --- | --- | --- | --- | --- | --- | --- | --- | --- | --- | --- | --- | --- | --- | --- | --- | --- | --- | --- | --- | --- | --- | --- | --- | --- | --- | --- | --- | --- | --- | --- | --- | --- | --- | --- | --- | --- | --- | --- | --- | --- | --- | --- | --- | --- | --- | --- | --- | --- | --- | --- | --- | --- | --- | --- | --- | --- | --- | --- | --- | --- | --- | --- | --- | --- | --- | --- | --- | --- | --- | --- | --- | --- | --- | --- | --- | --- | --- | --- | --- | --- | --- | --- | --- | --- | --- | --- | --- | --- | --- | --- | --- | --- | --- | --- | --- | --- | --- | --- | --- | --- | --- | --- | --- | --- | --- | --- | --- | --- | --- | --- | --- | --- | --- | --- | --- | --- | --- | --- | --- | --- | --- | --- | --- | --- | --- | --- | --- | --- | --- | --- | --- | --- | --- | --- | --- | --- | --- | --- | --- | --- | --- | --- | --- | --- | --- | --- | --- | --- | --- | --- | --- | --- | --- | --- | --- | --- | --- | --- | --- | --- | --- | --- | --- | --- | --- | --- | --- | --- | --- | --- | --- | --- | --- | --- | --- | --- | --- | --- | --- | --- | --- | --- | --- | --- | --- | --- | --- | --- | --- | --- | --- | --- | --- | --- | --- | --- | --- | --- | --- | --- | --- | --- | --- | --- | --- | --- | --- | --- | --- | --- | --- | --- | --- | --- | --- | --- | --- | --- | --- | --- | --- | --- | --- | --- | --- | --- | --- | --- | --- | --- | --- | --- | --- | --- | --- | --- | --- | --- | --- | --- | --- | --- | --- | --- | --- | --- | --- | --- | --- | --- | --- | --- | --- | --- | --- | --- | --- | --- | --- | --- | --- | --- | --- | --- | --- | --- | --- | --- | --- | --- | --- | --- | --- | --- | --- | --- | --- | --- | --- | --- | --- | --- | --- | --- | --- | --- | --- | --- | --- |
