# Supplementary material for: Prognostic implication of leucocyte subpopulations in diffuse large B-cell lymphoma
Source: Oncotarget. 2017 May 12;8(29):47790–800. doi: 10.18632/oncotarget.17830 (PMC5564605; doi:10.18632/oncotarget.17830)
Supplement: Supplementary file 3 [file oncotarget-08-47790-s003.docx]

**S2 Table. Multivariate analysis for overall survival**

| **Prognostic factors** | **Model 1** | | **Model 2** | | **Model 3** | | **Model 4** | |
| --- | --- | --- | --- | --- | --- | --- | --- | --- |
|  | **HR (95% CI)** | **P value** | **HR (95% CI)** | **P value** | **HR (95%CI)** | **P value** | **HR (95%CI)** | **P value** |
| lymphocytes(%) | 0.898(0.807-0.998) | 0.045 | 0.895(0.805-0.997) | 0.043 | 0.914(0.824-1.013) | 0.085 | 0.917(0.823-1.021) | 0.114 |
| NK&T lymphocytes(%) | 0.875(0.772-0.991) | 0.036 | 0.872(0.768-0.990) | 0.034 | 0.894(0.792-1.009) | 0.069 | 0.896(0.787-1.021) | 0.100 |
| CD16- non-cytotoxic NK&T lymphocytes(%) | 0.865(0.741-1.009) | 0.066 | 0.860(9.735-1.007) | 0.061 | 0.875(0.752-1.018) | 0.084 | 0.882(0.752-1.033) | 0.119 |
| monocytes(X10^9^) | 7.768(1.090-55.385) | 0.041 | 14.170(1.130-177.73) | 0.040 | 24.06(0.912-635.1) | 0.057 | 24.07(0.913-634.5) | 0.057 |
| CD16- monocytes(X10^9^) | 14.476(0.863-242.9) | 0.063 | 15.791(0.831-299.97) | 0.066 | 29.21(0.746-1144.3) | 0.071 | 29.48(0.749-1160.5) | 0.071 |
| CD16+ monocytes(X10^9^) | 559.3(4.547-6.9X104) | 0.010 | 9.59E6(3.708-2.48E13) | 0.033 | 3.05E9(15.51-6.00E17) | 0.025 | 2.53E9(11.27-5.67E17) | 0.027 |
| mature neutrophils(X10^9^) | 1.401(1.141-1.721) | 0.001 | 1.416(1.136-1.765) | 0.002 | 1.782(1.236-2.570) | 0.002 | 1.822(1.246-2.662) | 0.002 |
| mature neutrophils(%) | 1.047(0.983-1.116) | 0.152 | 1.049(0.984-1.118) | 0.147 | 1.037(0.972-1.106) | 0.276 | 1.036(0.971-1.105) | 0.280 |
| CD16-monocytes/CD16+monocytes | 0.887(0.788-0.999) | 0.048 | 0.887(0.787-1.000) | 0.050 | 0.885(0.792-0.989) | 0.031 | 0.857(0.759-0.967) | 0.012 |
| cytotoxic NK&T lymphocytes /CD16+ monocyte | 0.900(0.785-1.033) | 0.135 | 0.886(0.761-1.032) | 0.119 | 0.906(0.795-1.032) | 0.136 | 0.907(0.791-1.039) | 0.160 |
| mature neutrophils /cytotoxic NK&T lymphocytes | 1.014(1.004-1.024) | 0.008 | 1.018(1.005-1.031) | 0.006 | 1.014(1.002-1.027) | 0.019 | 1.018(1.003-1.034) | 0.019 |
| mature neutrophils /noncytotoxic NK&T lymphocytes | 1.017(0.994-1.041) | 0.156 | 1.017(0.991-1.043) | 0.198 | 1.003(0.975-1.032) | 0.822 | 0.983(0.931-1.037) | 0.526 |

Model 1 was adjusted by IPI

Model 2 was adjusted by IPI and bone marrow

Model 3 was adjusted by IPI, ECOG and bone marrow

Model 4 was adjusted by IPI, ECOG, number of extra nodal involvement and bone marrow
